# Triptolide (TPL) Inhibits Global Transcription by Inducing Proteasome-Dependent Degradation of RNA Polymerase II (Pol II)

**DOI:** 10.1371/journal.pone.0023993

**Published:** 2011-09-13

**Authors:** Ying Wang, Jin-jian Lu, Li He, Qiang Yu

**Affiliations:** 1 Department of Pharmacology, Shanghai Institute of Materia Medica, Chinese Academy of Sciences, Shanghai, People's Republic of China; 2 College of Life Sciences, Zhejiang Chinese Medical University, Hangzhou, People's Republic of China; French National Centre for Scientific Research, France

## Abstract

Triptolide (TPL), a key biologically active component of the Chinese medicinal herb *Tripterygium wilfordii* Hook. f., has potent anti-inflammation and anti-cancer activities. Its anti-proliferative and pro-apoptotic effects have been reported to be related to the inhibition of Nuclear Factor κB (NF-κB) and Nuclear Factor of Activated T-cells (NFAT) mediated transcription and suppression of HSP70 expression. The direct targets and precise mechanisms that are responsible for the gene expression inhibition, however, remain unknown. Here, we report that TPL inhibits global gene transcription by inducing proteasome-dependent degradation of the largest subunit of RNA polymerase II (Rpb1) in cancer cells. In the presence of proteosome inhibitor MG132, TPL treatment causes hyperphosphorylation of Rpb1 by activation of upstream protein kinases such as Positive Transcription Elongation Factor b (P-TEFb) in a time and dose dependent manner. Also, we observe that short time incubation of TPL with cancer cells induces DNA damage. In conclusion, we propose a new mechanism of how TPL works in killing cancer. TPL inhibits global transcription in cancer cells by induction of phosphorylation and subsequent proteasome-dependent degradation of Rpb1 resulting in global gene transcription, which may explain the high potency of TPL in killing cancer.

## Introduction

The Chinese medicinal herb *Tripterygium wilfordii* Hook. f. has been used for centuries to treat inflammation, autoimmune diseases, and cancers [1], [2]. Triptolide (TPL), a diterpenoid triepoxide, is one of the major biologically active components of the herb. Many studies have revealed that TPL has a myriad of biological functions, including immunosuppression, anti-inflammation, and anti-cancer, since its first isolation in 1972 [1], [3], [4], [5]. Although the activity of TPL in inducing tumor cell death has been well documented, its modes of action have not been thoroughly elucidated. TPL has been mostly reported to reduce mRNA levels of many genes. It has been reported to strongly inhibit transcription of numerous pro-inflammatory mediators [6]. It is a potent inhibitor of NF-κB and NFAT-mediated transcription [7]. It is also an inhibitor of the transcription of HSP 70 [8]. Recently, there have been reports demonstrating that TPL has global transcriptional inhibitory activities [9], [10], [11]. The precise mechanism of TPL, however, is not well characterized. Based on these observations, it is reasonable to speculate that a global transcriptional inhibition may be the key for TPL to exert its potent antitumor activities against a broad range of human cancers.

RNA polymerase II (Pol II) is a large protein complex of 12 subunits. It is involved not only in transcription but also in other biological processes in the nucleus, including mRNA processing and transcription-coupled repair (TCR) [12], [13], [14]. The largest subunit, Rpb1, contains a unique C-terminal domain (CTD) that consists of the heptapeptide Tyr-Ser-Pro-Thr-Ser-Pro-Ser repeated 52 times in mammalian cells [12], [14], [15]. Pol II undergoes post-translational modifications, such as phosphorylation and ubiquitination [16], [17]. Phosphorylation of the CTD of Rpb1 has specialized roles. First, CTD undergoes a cycle of phosphorylation/dephosphorylation during the transcription cycle [18], [19]. At the beginning of transcription, a hypophosphorylated form of Pol II (IIA) preferentially binds to promoters. Later on, promoter clearance and transcript elongation are associated with phosphorylation of the CTD to yield a hyperphosphorylated form (IIO). Second, the phosphorylated CTD plays an important role in mRNA processing because many proteins involved in pre-mRNA processing bind to the phosphorylated CTD [14], [20]. Third, many studies reveal that DNA damage by UV or hydrogen peroxide (H_2_O_2_) induces phosphorylation of CTD of Rpb1 accompanied with ubiquitination and subsequent proteasome degradation. When the DNA lesions are located on the transcribed strand of a gene, cells trigger TCR to remove the lesion. In TCR, the progression of Pol II is blocked at the DNA lesions within the coding region. Stalled Pol II needs to be removed from the site of damage in order for the repair machinery to assemble, possibly by phosphorylation-mediated proteolysis [12], [17], [21]. Several kinases such as CDK7, CDK8, CDK9 (a component of P-TEFb), ERK and JNK have been shown to be responsible for the phosphorylation of CTD under different conditions [12], [14], [22], [23], [24].

In our study, it was found that TPL inhibited global gene transcription, which was consistent with a recent discovery [9], [11]. Detailed studies revealed that TPL induced phosphorylation and subsequent proteasome degradation of Rpb1 in a dose and time-dependent manner in cancer cell lines. The protein kinase P-TEFb is required for the TPL-induced phosphorylation of Rpb1. We also observed DNA damage induced by TPL using comet assay. We propose that DNA damage-trigged phosphorylation and degradation of Rpb1 may be the key for a global transcriptional inhibition by TPL. Our study uncovered a new mechanism of TPL in inducing cancer cell death.

## Results

### TPL inhibited global transcription in cancer cells

**Figure 1:**
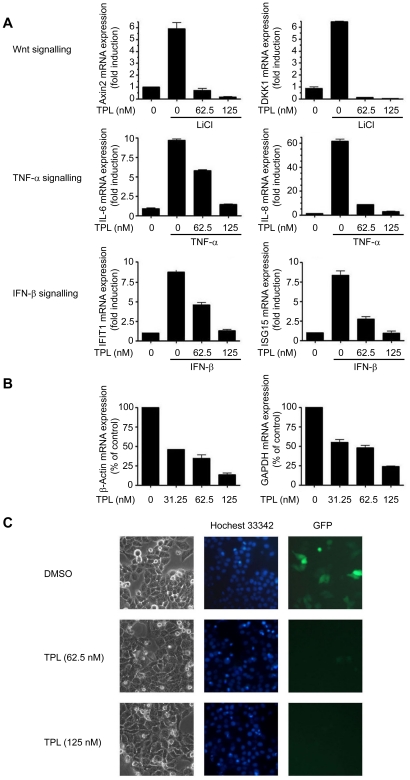
TPL inhibited global transcription. (A) Effects of TPL on the transcription of inducible genes. Hela cells were pretreated with TPL at indicated concentrations for 2 h before stimulation by LiCl (20 mM), TNF-α (100ng/ml) or IFN-β (100ng/ml) for another 3 h. The mRNA expression of the indicated genes was analyzed by Real-time PCR. All values were normalized to the mRNA level of β-Actin because the mRNA level of β-Actin was not affected at this time point. (B) Effects of TPL on the transcription of housekeeping genes. Hela cells were treated with TPL for 12 h. The mRNA expression of the indicated genes was analyzed by Real-time PCR. Equal amounts of RNA were used to normalize the loadings. (C) Effects of TPL on expression of transiently transfected genes. Hela cells were transiently transfected with a pEGFP-N1 plasmid. 6 h after transfection, the medium was replaced with the fresh medium containing TPL at indicated concentrations and the Hela cells were incubated for another 15h. GFP expression was visualized by fluorescent microscopy. The picture is a representative of four visual fields. Original magnification: ×200.

In an attempt to understand the molecular mechanisms of TPL in inducing cancer cell death, we used Real-time PCR to examine the effects of TPL on the transcription of several inducible and housekeeping genes. The results showed that short-time treatment of TPL in Hela cells strongly suppressed Wnt, TNF-α or IFN-β induced target genes transcription (Fig. 1A). Similar inhibitory effects were observed on housekeeping gene β-Actin and GAPDH when Hela cells were treated with TPL for 12 h (Fig. 1B). We also examined the effect of TPL on transcription of a transiently transfected gene pEGFP-N1 in Hela cells. 6 h after transfection, the Hela cells were treated with TPL for another 15h. We found that GFP expression was dramatically inhibited by TPL (Fig. 1C). Taken together, these data strongly suggested that TPL inhibited global transcription.

### TPL reduced Rpb1 protein level in cancer cell lines

**Figure 2:**
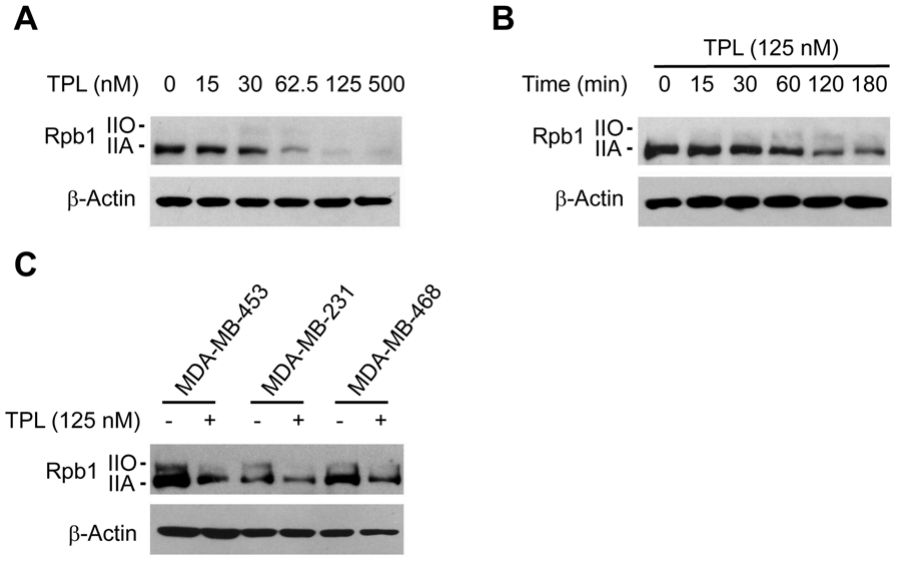
Effects of TPL on Rpb1 protein levels. (A) Hela cells were treated with TPL at indicated concentrations for 3 h. Whole cell lysates were processed for SDS-PAGE and Western blot analysis using antibodies as indicated. (B) Hela cells were treated with 125 nM TPL for various lengths of time (0–180 min) as indicated. Whole cell lysates were subjected to SDS-PAGE and Western blot analysis. (C) MDA-MB-453, MDA-MB-231 or MDA-MB-468 cells were treated with 125 nM TPL for 3 h. Whole cell lysates were subjected to SDS-PAGE and Western blot analysis. β-Actin served as a loading control.

To understand how TPL may affect global transcription, we analyzed the effects of TPL on the components of RNA polymerase II, because Pol II plays a crucial role in transcription. We first investigated the effects of TPL on the largest subunit of Pol II, Rpb1, by immunoblotting. We found that TPL reduced the protein level of Rpb1 in a dose-dependent manner in Hela cells (Fig. 2A). The decrease of Rpb1 protein level was evident at 1 h incubation with 125 nM of TPL, and reached maximum at 2 h (Fig. 2B). We also examined the effect of TPL on Rpb1 in other cancer cell lines, MDA-MB-453, MDA-MB-231 and MDA-MB-468 (Fig. 2C). Similar effects were observed when these cells were incubated with TPL for 3 h. All these results demonstrated that TPL reduced the protein level of Rpb1 in cancer cells.

### TPL induced phosphorylation and subsequent proteasome-dependent degradation of Rpb1

**Figure 3:**
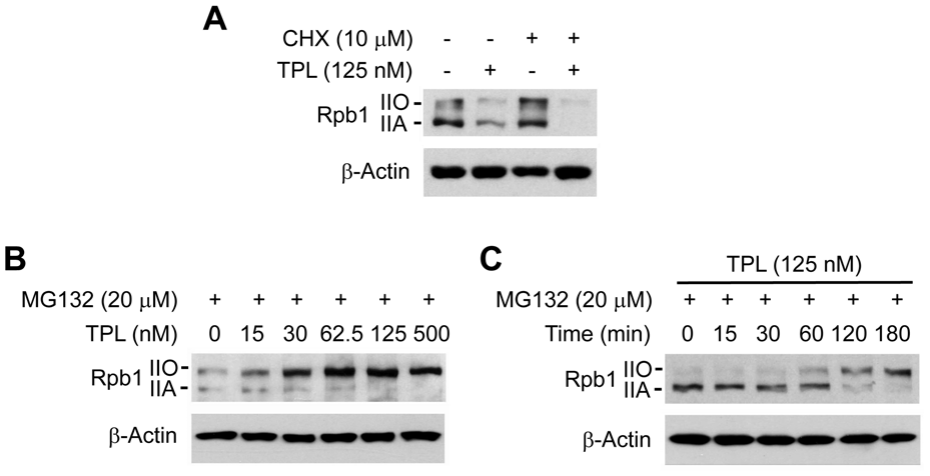
TPL induced phosphorylation and proteasome-dependent degradation of Rpb1. (A) Hela cells were pretreated with CHX (10 µM) for 0.5 h before addition of TPL (125 nM) and treated for another 3 h. Whole cell lysates were processed for Western blot analysis using antibodies as indicated. (B) Hela cells were pretreated with MG132 (20 µM) for 1 h and then treated with TPL at indicated concentrations for another 3 h. Whole cell lysates were subjected to SDS-PAGE and Western blot analysis. (C) Hela cells were treated with 125 nM TPL for various lengths of time (0–180 min) in the presence of MG132 (20 µM). Whole cell lysates were subjected to SDS-PAGE and Western blot analysis. β-Actin served as a loading control.

To address whether the reduction of Rpb1 protein level by TPL is due to inhibition of protein synthesis or induction of protein degradation, we examined the effects of TPL on Rpb1 protein level in the presence of protein synthesis inhibitor cycloheximide (CHX) or proteasome inhibitor MG132. The results showed that TPL still reduced Rpb1 protein levels in the presence of CHX (Fig. 3A). On the contrary, the decrease of Rpb1 protein level by TPL was inhibited by the MG132 treatment (Fig. 3B), suggesting that TPL reduced Rpb1 protein levels through a proteasome-dependent protein degradation mechanism. Furthermore, we found that the recovered Rpb1 was mostly in the phosphorylated form (Pol IIO) when the TPL-induced proteasomal degradation of Rpb1 was inhibited by MG132, and the phosphorylation of Rpb1 induced by TPL was also in a dose and time dependent manner (Fig. 3B and 3C). These results indicated that TPL induced phosphorylation of Rpb1 and the phosphorylated Rpb1 subsequently underwent proteasome-dependent degradation.

### TPL increased phosphorylation of Rpb1 by activating protein kinases

**Figure 4:**
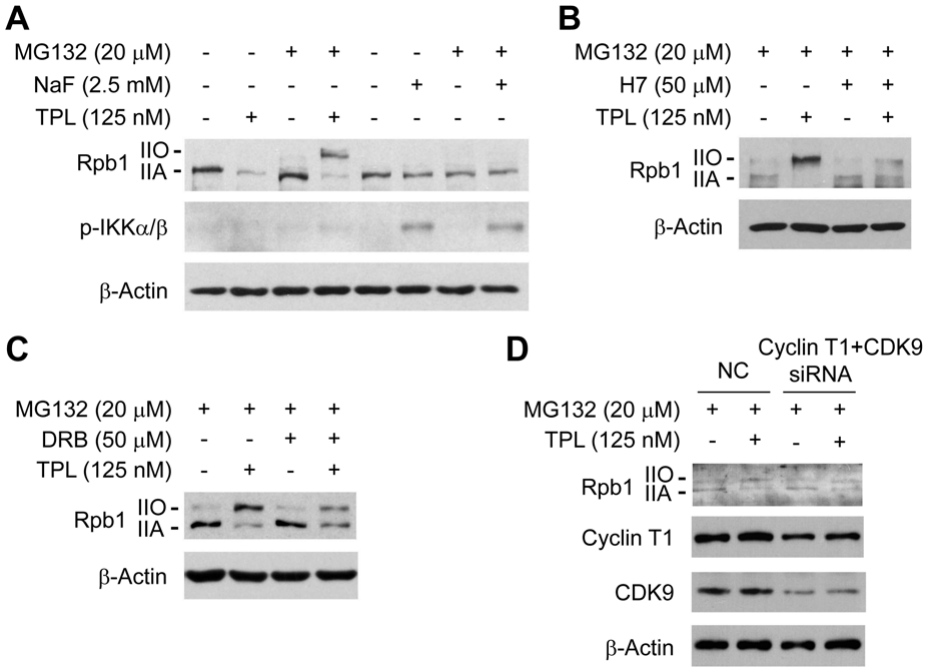
TPL induced phosphorylation of Rpb1 by activation of protein kinases. (A) Comparison of the effect of TPL with NaF on Rpb1. Hela cells were treated with TPL or NaF for 3 h in the presence/absence of MG132. Whole cell lysates were processed for SDS-PAGE and Western blot analysis using antibodies as indicated. (B) Hela cells were pretreated with MG132 for 1 h, and then treated with TPL in the presence/absence of 50 µM H7 for 3 h. Whole cell lysates were subjected to SDS-PAGE and Western blot analysis. (C) Hela cells were pretreated with MG132 for 1 h, and then treated with TPL in the presence/absence of 50 µM DRB for 3 h. Whole cell lysates were subjected to SDS-PAGE and Western blot analysis. (D) Analysis of effect of CDK9 and Cyclin T1 siRNA on TPL-induced Rpb1 phosphorylation. Hela cells were transfected with NC (negative control) or CDK9 and Cyclin T1 siRNA. 48 h after transfection, the Hela cells were pretreated with 20 µM MG132 for 1 h. Then the cells were treated with DMSO or 125 nM TPL for another 3 h before collected to SDS-PAGE and Western blot analysis. β-Actin served as a loading control.

We further investigated whether the increased phosphorylation of Rpb1 induced by TPL is caused by kinase activation or phosphatase inactivation. We first compared the effect of TPL with that of the general serine/theronine phosphatase inhibitor, NaF, on Rpb1.We found that different from the effect of TPL, inhibition of phosphatase by NaF treatment did not increase the phosphorylation level of Rpb1 in the presence of MG132 (Fig. 4A), suggesting that the increased phosphorylation of Rpb1 induced by TPL was not due to phosphatase inactivation. In addition, we examined the phosphorylation of IKK α/β as a positive control of phosphatase inhibition by NaF. We found that TPL did not increase the phosphorylation of IKK α/β, suggesting that the phosphorylation of Rpb1 by TPL was specific (Fig. 4A).

Next, we determined if the TPL-induced phosphorylation of Rpb1 was due to kinase activation by using a general protein kinase inhibitor H7. It was found that the phosphorylation of Rpb1 induced by TPL was inhibited by H7 when the proteasomal degradation of Rpb1 was blocked by MG132 (Fig. 4B), suggesting that TPL induced phosphorylation of Rpb1 by activating kinases.

To identify the exact kinase responsible for the TPL-induced phosphorylation, we used several specific kinase inhibitors targeting ERK, JNK or P-TEFb which are reported to phosphorylate Rpb1 [12], [25]. Only the P-TEFb inhibitor DRB partially inhibited the phosphorylation of Rpb1 (Fig. 4C) while the other two inhibitors had no effects on phosphorylation of Rpb1 (data not shown). P-TEFb is a complex of CDK9 and Cyclin T1. We further investigated the role of P-TEFb in TPL-induced Rpb1 phosphorylation by transfection of siRNAs of CDK9 and Cyclin T1 into Hela cells. The data demonstrated that the TPL-induced phosphorylation of Rpb1 was decreased when the protein levels of CDK9 and Cyclin T1 were reduced (Fig. 4D), which is consistent with result of the pharmacological inhibition of P-TEFb. These data strongly suggested that TPL-induced Rpb1 phosphorylation was regulated by P-TEFb.

### TPL induced DNA damage

**Figure 5:**
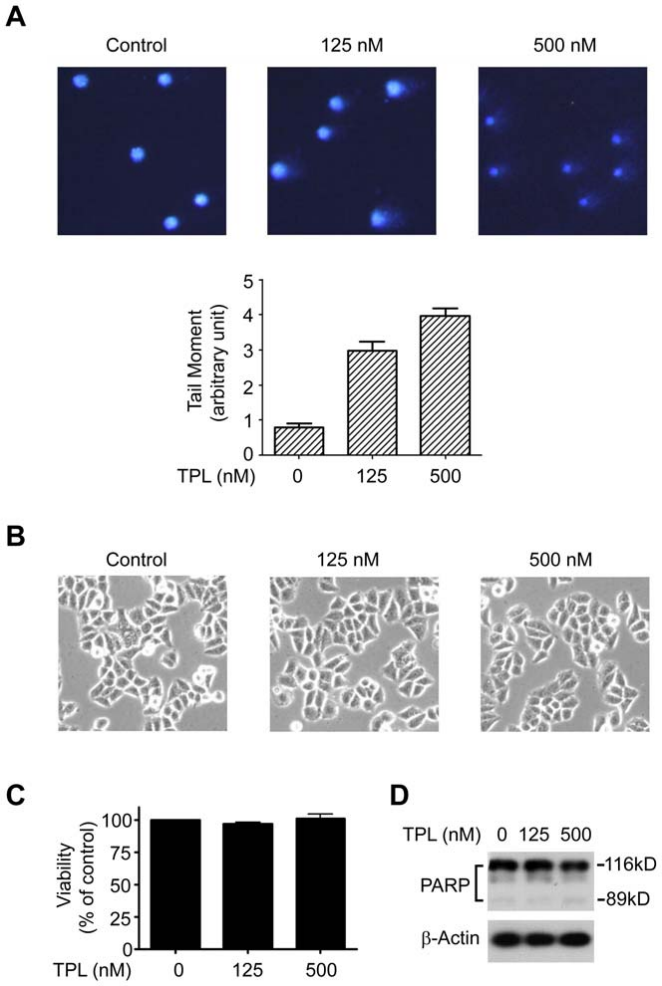
TPL induced DNA damage. (A) Comet assay. Hela cells were treated with TPL at indicated concentrations for 3 h, and then subjected to comet assay. Quantification analysis of the results was presented below the graph. The pictures are representatives of four fields. Original magnification: ×200. (B) Morphology of Hela cells treated with TPL at indicated concentrations for 3 h. The pictures are representatives of four fields. Original magnification: ×200. (C) Viability of Hela cells treated with TPL at indicated concentrations for 3 h. (D) Apoptosis analysis of Hela cells treated with TPL at indicated concentrations for 3 h. Whole cell lysates were processed for Western blot analysis using anti-PARP antibody. β-Actin served as a loading control.

It is reported that UV or H_2_O_2_-induced DNA damage leads to phosphorylation and subsequent proteasomal degradation of Rpb1 to allow TCR to repair the DNA lesion [12], [14]. We therefore investigated whether the TPL-induced phosphorylation and degradation of Rpb1 was due to DNA damage. We examined the effect of TPL on DNA damage by comet assay, which is also known as single cell gel electrophoresis assay to detect DNA damage in a single cell. The overall structure of genome DNA resembles a comet with a circular head corresponding to the undamaged DNA and a tail of damaged DNA. The brighter and longer the tail is, the higher the level of the damage is [26]. As shown in Fig. 5A, 3 h-treatment of TPL in Hela cells induced DNA damage at 125 nM while more comet tails appeared at 500 nM (Fig. 5A), suggesting that TPL induced DNA damage. At this time point, TPL had no obvious effects on the morphology as well as apoptosis of Hela cells (Fig. 5B–5D), ruling out the possibility that comet tails caused by TPL were the result of apoptosis.

### Discussion

*Tripterygium wilfordii* Hook. f. has long been used as a folk medicine to treat cancer in China. TPL is one of the major active components of the plant, which possesses strong anti-cancer activity against leukemias, lymphomas, as well as solid tumors [27]. The mode of action of its anti-neoplastic effect is complex and not well-characterized. Thus, elucidating the molecular mechanism of TPL in killing cancer cells will help us to understand and to better use this traditional Chinese medicine in cancer therapy.

Many studies have been carried to elucidate the mode of action of TPL in inducing cancer cell death. The conclusions are however diverse. It has been reported that its pro-apoptotic activity is due to modulation of apoptosis-activating proteins [28], [29] and is partially dependent on calcium [30]. It was found that cancer cell-specific enhancement of cisplatin-induced cytotoxicity with TPL was through an interaction of inactivated glycogen synthase kinase-3 with p53 [31]. It was also reported that TPL suppressed NF-κB, AP-1, NFAT, and HSF1 transactivation, and the inhibition of NF-κB and HSF1 was at a step following their DNA binding [7], [32], [33]. In contrast to above studies, which all point to certain specificities of TPL, TPL has also been reported to suppress general transcription by altering nuclear substructure [9]. It appeared from these studies that TPL influences many proteins and pathways that associated with cell growth and survival. The direct targets of TPL, however, remain unidentified.

We observed several effects of TPL on cancer cells in our study. First, TPL inhibited global transcription in cancer cells. Second, short-time treatment of TPL induced a proteasome-dependent degradation of Rpb1, the largest subunit of RNA polymerase II. Third, TPL increased phosphoryalation of Rpb1 by activating protein kinases such as P-TEFb. Fourth, short-time treatment of TPL induced DNA damage in cancer cells.

It is not clear how the TPL-induced DNA damage may activate P-TEFb. However, it is known that P-TEFb is a major kinase of Rpb1, and cells trigger TCR to activate kinases to specifically repair DNA lesions located in the transcribed strand of DNA in the UV or H_2_O_2_-induced DNA damage [12], [14]. During TCR, Rpb1 undergoes phosphorylation which signals for proteasome-dependent degradation. The effects of TPL on Rpb1 seem similar to those of UV which caused P-TEFb activation as well as subsequent phosphorylation and degradation of Rpb1. It is logic to hypothesize that DNA is the primary target of TPL. TPL causes DNA damage, which in turn triggers TCR, then activates certain protein kinases, such as P-TEFb, to phosphorylate Rpb1. The phosphorylated Rpb1 is subsequently targeted to proteosome to be degraded. The transcription of the targeted cells is therefore inhibited, which ultimately causes cancer cells to die. The dose of TPL to cause DNA damage is higher than that to induce phosphorylation of Rpb1 because of the sensitivity of the comet assay. It requires a large amount of DNA damage, therefore a high dose of TPL, to give rise to a visible effect. The Rpb1 phosphorylation assay however is far more sensitive than the comet assay, which is the reason why the doses of TPL were different in the two assays.

We do not think the DNA damage we observed is the result of cell apoptosis because we did not observe apoptosis when we observed the DNA damage. Our experiments also exclude the possibility that TPL may induce ROS to cause the degradation of Rpb1 (data not shown). It has been reported that compounds posses epoxy groups are reported to alkylate DNA and the epoxy groups are responsible for this reactivity [34]. There are three epoxy groups in TPL which may alkylate DNA and result in DNA damage, although we have not obtained direct evidences to support our hypothesis.

In summary, our study discovered a new mode of action of TPL in anti-cancer therapy. TPL inhibited global transcription by inducing DNA damage and Rpb1 degradation in cancer cells. Global transcriptional inhibition induces apoptosis in cancer cells [35], [36], . Our discovery may explain the high efficacy of TPL against a wide range of cancers. As the mode of action of TPL continues to unfold, we will eventually understand the multiple regulatory actions of TPL on cancer cells as well as its therapeutic potentials.

## Materials and Methods

### Cell Lines and Culture

All the cancer cell lines used, cervix cancer cell line Hela and breast cancer cell lines MDA-MB-231, MDA-MB-453 and MDA-MB-468, were obtained from the American Type Culture Collection (ATCC) and were cultured in DMEM containing 10% calf serum and 1% penicillin-streptomycin.

### Reagents

Triptolide, proteasome inhibitor MG132, and protein synthesis inhibitor cycloheximide (CHX) were purchased from Calbiochem. Protein kinase inhibitor H7, P-TEFb inhibitor DRB, and serine/theronine phosphatase inhibitor NaF were purchased from Sigma.

### Plasmids and Transfection

pEGFP-N1 was obtained from Clonetech and transient transfection of Hela cells with pEGFP-N1 was performed using Lipofectamine 2000 (Invitrogen).

### Western Blot Analysis

Whole cell lysates were prepared in 1× Laemmli sample buffer (Sigma) to extract total proteins. Equivalent amounts of total cellular protein were resolved by a 7.5% SDS- PAGE and transferred onto nitrocellulose membranes (Millipore). The membranes were blocked in 5% nonfat milk in TBS containing 0.1% Tween 20 (TBST) for 1 h at room temperature, and then incubated with primary antibodies in 5% bovine serum albumin (BSA) in TBST at 4°C overnight. The membranes were then washed with TBST and incubated with horseradish peroxidase-conjugated secondary antibody in 5% BSA in TBST for 1 h at room temperature. Immune complexes were detected by enhanced chemiluminescence (Pierce).

Antibodies used in Western blotting were rabbit anti-Rpb1, rabbit anti-Cyclin T1and rabbit anti-CDK9 (Santa Cruz Biotechnology); rabbit anti-phospho-IKK α/β and rabbit anti-PARP (Cell Signaling Technology); mouse anti-β-Actin (Sigma).

### Real-time PCR

Total cellular RNA was isolated with TRIzol (Invitrogen) according to the manufacturer's instructions. Reverse transcription of purified RNA was performed using oligo(dT) primer. The quantification of gene transcripts performed by real-time PCR using SYBR Green I dye (Invitrogen). All values of inducible genes were normalized to the level of β-Actin mRNA. For accessing the mRNA levels of the housekeeping genes β-Actin and GAPDH, equal amounts of RNA were used for their normalization.

### siRNA knockdown

Small interfering RNAs (siRNAs) for the negative control (NC), CDK9 and Cyclin T1 were purchased from GenePharma and transfected using lipofetamine 2000 (Invitrogen). The sequences of the siRNA oligonucleotides used in this study were as follows: NC siRNA, 5-UUCUCCGAACGUGUCACGUTT-3; CDK9 siRNA, 5-UAGGGACAUGAAGGCUGCUAA-3; Cyclin T1 siRNA, 5-UCCCUUCCUGAUACUAGA A-3. Hela cells were plated in 6-well plates. At 40% confluence, 150 pmol of NC siRNA or 75 pmol CDK9 and 75 pmol Cyclin T1 siRNA were transfected into cells. 48 h posttransfection, the Hela cells were pretreated with 20 µM MG132 for 1 h. Then the cells were treated with DMSO or 125 nM TPL for another 3 h before collected to Western blot analysis. Efficiency of protein knock down was determined at this time point.

### Alkaline single-cell gel electrophoresis assay (comet assay)

The alkaline single-cell gel electrophoresis assay was performed as previously described [38], [39]. Briefly, HeLa cells were treated with TPL for 3 h before being pelleted and resuspended in ice-cold PBS. Resuspended cells were then mixed with prewarmed 1% low–melting point agarose. The agarose-cell mixture was placed on a slide pre-coated with 0.5% agarose and spread gently with a cover slip. After 15 min at 4^o^C, the slides were immersed in precooled lysis solution (2.5 M NaCl, 100 mM Na_2_EDTA, 10 mM Tris-HCl (pH 10), 10% DMSO, 1% Triton X-100, and 1% laurosylsarcosinate) for 90 min in a dark chamber. After soaking with electrophoresis buffer (0.3 M NaOH and 1 mM EDTA) for 20 min, the slides were subjected to electrophoresis for 20 min. Finally, the cells were stained with DAPI. Cells were viewed using an Olympus BX51 fluorescence microscope and quantitation was achieved by analyzing at least 50 randomly selected comets per slide with the Komet 5.5 software (Kinetic Imaging Ltd., Nottingham, UK) using the parameter olive tail moments (arbitrary units, defined as the product of the percentage of DNA in the tail multiplied by the tail length).

### Determination of cell viability

Hela cells were treated with TPL for 3 h and then cell viability was determined by 3-(4, 5-Dimethylthiazol-2-yl)-2, 5-Diphenyltetrazolium Bromide (MTT) Assay as described [40].
